# Genetics of Autosomal Recessive Polycystic Kidney Disease and Its Differential Diagnoses

**DOI:** 10.3389/fped.2017.00221

**Published:** 2018-02-09

**Authors:** Carsten Bergmann

**Affiliations:** ^1^Center for Human Genetics, Bioscientia, Ingelheim, Germany; ^2^Department of Medicine, University Hospital Freiburg, Freiburg, Germany

**Keywords:** autosomal recessive polycystic kidney disease (ARPKD), ADPKD, PKHD1, DZIP1L, PKD1/PKD2, ciliopathies, HNF1β/TCF2, nephronophthisis (NPHP)

## Abstract

Autosomal recessive polycystic kidney disease (ARPKD) is a hepatorenal fibrocystic disorder that is characterized by enlarged kidneys with progressive loss of renal function and biliary duct dilatation and congenital hepatic fibrosis that leads to portal hypertension in some patients. Mutations in the *PKHD1* gene are the primary cause of ARPKD; however, the disease is genetically not as homogeneous as long thought and mutations in several other cystogenes can phenocopy ARPKD. The family history usually is negative, both for recessive, but also often for dominant disease genes due to *de novo* arisen mutations or recessive inheritance of variants in genes that usually follow dominant patterns such as the main ADPKD genes *PKD1* and *PKD2*. Considerable progress has been made in the understanding of polycystic kidney disease (PKD). A reduced dosage of disease proteins leads to the disruption of signaling pathways underlying key mechanisms involved in cellular homeostasis, which may help to explain the accelerated and severe clinical progression of disease course in some PKD patients. A comprehensive knowledge of disease-causing genes is essential for counseling and to avoid genetic misdiagnosis, which is particularly important in the prenatal setting (e.g., preimplantation genetic diagnosis/PGD). For ARPKD, there is a strong demand for early and reliable prenatal diagnosis, which is only feasible by molecular genetic analysis. A clear genetic diagnosis is helpful for many families and improves the clinical management of patients. Unnecessary and invasive measures can be avoided and renal and extrarenal comorbidities early be detected in the clinical course. The increasing number of genes that have to be considered benefit from the advances of next-generation sequencing (NGS) which allows simultaneous analysis of a large group of genes in a single test at relatively low cost and has become the mainstay for genetic diagnosis. The broad phenotypic and genetic heterogeneity of cystic and polycystic kidney diseases make NGS a particularly powerful approach for these indications. Interpretation of genetic data becomes the challenge and requires deep clinical understanding.

## Polycystic Kidney Disease and Ciliary Dysfunction

Polycystic kidney disease (PKD) is a clinically and genetically heterogeneous group of disorders with phenotypes ranging from manifestation *in utero* to clinically silent disease well into adulthood. PKD can be inherited in a dominant (ADPKD) and recessive (ARPKD) way. In addition to ADPKD and ARPKD, cystic kidney disease is a common feature of syndromic ciliopathies. Patients with ciliopathies represent a significant proportion of all patients with end stage renal disease (ESRD). In addition to fibrocystic renal changes, patients often present with extrarenal manifestations (1–7). Cystic kidneys require careful clinical workup to identify the underlying genetic disorder, especially in children. In recent years, our understanding of the mechanistic basis of polycystic kidney disease has increased substantially. It has been shown that PKD-associated proteins co-localize in multimeric complexes at distinct subcellular epithelial sites. Ultrastructural analysis reveals that PKD-associated proteins localize to the primary cilium and suggests that dysfunctional primary cilia may be the source of the underlying etymology of cystic kidney disease (1–4, 8). Cilia are microtubule-based organelles that project from the surface of most quiescent vertebrate cells, and function in chemo- and mechanosensation and fluid transport. The ciliary axoneme is anchored in the cell by the centriole-derived basal body and is ensheathed by the ciliary membrane. Located between the basal body and axoneme, the transition zone (TZ) forms a diffusion barrier that modulates the selective passage of proteins to and from the cilium (9–12). The insights gained in recent years have led to approval of Tolvaptan (Jinarc/Samsca) for ADPKD in many countries and also to the establishment of multiple clinical trials evaluating emerging therapeutic strategies that may have the potential for rational personalized therapies in future years (13).

An accurate diagnosis is essential both in the management of patients with cystic kidneys and in counseling their families. When an effort is made to classify the wide array of different entities with renal cysts, knowledge about the family history, the clinical phenotype, location and morphology of the cysts, and any possible extrarenal manifestations should help in making a diagnosis. Conventional heritable cystic kidney diseases include ADPKD and ARPKD, as well as nephronophthisis (NPHP), medullary cystic kidney disease, and glomerulocystic kidney disease. Other rare ciliopathies that may affect multiple tissues need to be taken into consideration.

## Prevalence and Recurrence Risk

ARPKD occurs in about 1 in 20,000 live births among Caucasians. This corresponds to a carrier frequency of approximately 1:70 in non-isolated populations (14–16). The exact incidence is unknown since published studies vary in the cohorts of patients examined (e.g., autopsied patients vs. moderately affected patients followed by pediatricians), and some severely affected babies may die perinatally without a definitive diagnosis. Isolated or inbred populations with many consanguineous marriages may have much higher prevalence, e.g., Kääriäinen reported an incidence of 1:8,000 in Finland (17). Notably, among children with polycystic kidneys, the total number of patients with early-onset ADPKD may be comparable to those of children with ARPKD.

Given its autosomal recessive mode of inheritance, the recurrence risk for subsequent pregnancies of parents of an affected child is 25%. Males and females are equally affected. As indicated by formal genetics, unaffected siblings harbor a two-thirds risk of being a carrier for ARPKD. By definition, heterozygous carriers do not show any clinical disease manifestations, however may have an increased risk for polycystic liver disease (PLD). Healthy siblings, other relatives, and patients themselves seeking genetic counseling for their own family planning can usually be reassured of a low risk for offspring with ARPKD when neither the partner is related with the index family nor a case of ARPKD is known in the partner’s pedigree. At an assumed heterozygosity rate of 1:70, the estimated risk is 1:140 for offspring of patients, 1:420 for offspring of patients’ healthy siblings, and 1:560 for offspring of patients’ healthy uncles/aunts.

## Clinical Course

**Figure 1:**
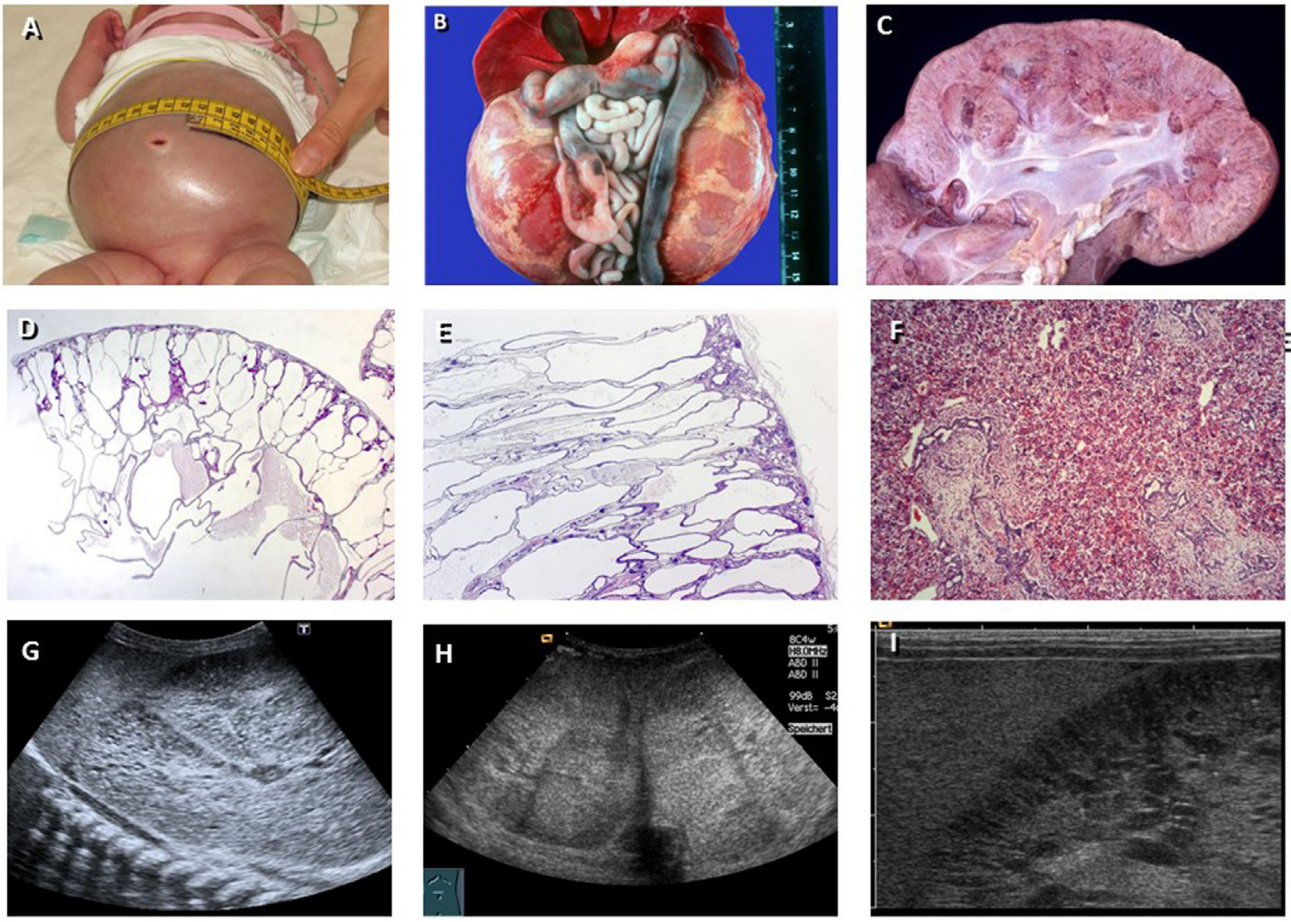
Autosomal recessive polycystic kidney disease (ARPKD). **(A)** Baby with distended abdomen due to voluminous kidneys that lead to respiratory problems. **(B)** Abdominal situs of a perinatally demised ARPKD patient with symmetrically enlarged kidneys that maintain their reniform configuration. **(C)** Cross section of an ARPKD kidney with cortical extension of fusiform and cylindrical spaces arranged radially throughout the renal parenchyma from medulla to cortex. **(D,E)** Microscopically, fusiform dilations of renal collecting ducts and distal tubuli lined by columnar or cuboidal epithelium. These dilated collecting ducts run perpendicular to the renal capsule. **(F)** Obligatory hepatobiliary changes in ARPKD known as ductal plate malformation characterized by dysgenesis of the hepatic portal triad with hyperplastic biliary ducts and congenital hepatic fibrosis. **(G–I)** Renal ultrasound of young children with ARPKD demonstrating enlarged echogenic kidneys with fusiform dilations of collecting ducts and distal tubules arranged radially throughout the renal parenchyma from medulla to cortex.

Clinically, ARPKD typically presents much earlier and is more severe than ADPKD. The majority of cases are identified at birth or late in pregnancy. Affected fetuses display a “Potter” phenotype with massively enlarged kidneys, pulmonary hypoplasia, a characteristic facies, and contracted limbs with club feet. Approximately 30–50% of affected neonates die shortly after birth from respiratory insufficiency due to pulmonary hypoplasia and thoracic compression by the excessively enlarged kidneys. However, the clinical spectrum is often more variable than generally presumed. While the majority of cases are identified at birth or late in pregnancy, there have been some reported cases of elderly people with ARPKD being only moderately affected (18, 19) (Figure 1).

Ultrasound scans typically show bilaterally enlarged hyperechoic kidneys with poor corticomedullary differentiation, retained reniform contour, and multiple tiny cysts confined to distal tubules and collecting ducts (Figure 1). With advancing clinical course, the kidney structure might increasingly resemble the pattern observed in ADPKD with renal cysts that vary considerably in size and appearance, often also accompanied by some degree of interstitial fibrosis (20). Arterial hypertension, often difficult to control despite multi-drug treatment, often develops during the first months of life and affects up to 80% of children with ARPKD. Close monitoring of blood pressure is essential as hypertension can exacerbate already deteriorating renal function (19).

In addition to renal manifestations and other comorbidities which characterize the early stages of ARPKD, patients necessarily develop some form of histologic liver involvement explaining why the entity is also entitled “Polycystic Kidney and Hepatic Disease 1 (PKHD1).” These histological changes are a result of defective remodeling of the ductal plate leading to biliary duct ectasia and hepatic fibrosis collectively known as ductal plate malformation (DPM) (7, 21) (Figure 1F). DPM is also a common feature of other ciliopathies. Biliary anomalies may develop at any stage of the physiologic involution-remodeling process, and the timing or stage of development determines the resulting clinical and histological phenotype (22).

Outside of the portal tracts, the remaining structures of the liver develop normally in ARPKD. In contrast to similar diseases, such as *HNF1β*, enzyme levels are typically within normal ranges, with the exception of cholestasis parameters, which may be elevated. In older patients, hepatobiliary complications are more significant causes of morbidity. DPM leads to progressive fibrosis of the liver resulting in portal hypertension. This can lead to a number of complications such as hypersplenism and esophageal varices (15, 19). ARPKD is one of the two major indications for combined liver and kidney transplantation during childhood. The best timing and strategy for combined transplantation is still a matter of debate and usually requires individualized decision-making on a case by case basis. Moreover, there is some preliminary evidence that adult ARPKD patients beyond the age of 40 years may have a slightly increased risk to develop hepatic tumors, especially cholangiocarcinoma (21, 23). While a preponderance of ARPKD patients show a consistent disease progression, individual patients may present with atypical phenotypes, such as exclusive or predominant phenotypes of either the liver or kidneys. Accordingly, in certain cases of isolated congenital hepatic fibrosis (CHF) (Caroli’s disease), *PKHD1* has been demonstrated to be the causative gene (24). Similarly, in mouse models for *Pkhd1*, the liver phenotype is stronger than renal involvement (25–28). Overall, ARPKD is associated with a significant portion of liver and kidney associated morbidity in children, resulting in diminished life expectancy.

## *PKHD1* Gene and Its Encoded Protein Polyductin/Fibrocystin

In 2002, *PKHD1* was identified as the main gene for ARPKD (29, 30). *PKHD1* is among the largest disease genes in the human genome, extending over a genomic segment of at least 470 kb and including a minimum of 86 exons. Both *PKHD1* and its murine ortholog undergo a complex and extensive pattern of alternative splicing, generating transcripts highly variable in size. In accordance with the disease phenotype, the gene is highly expressed in fetal and adult kidney and at lower levels in liver (30, 31). Weak expression is present in other tissues too, including pancreas and arterial wall. The longest *PKHD1* transcript contains 67 exons encoding a protein of 4,074 amino acids.

**Figure 2:**
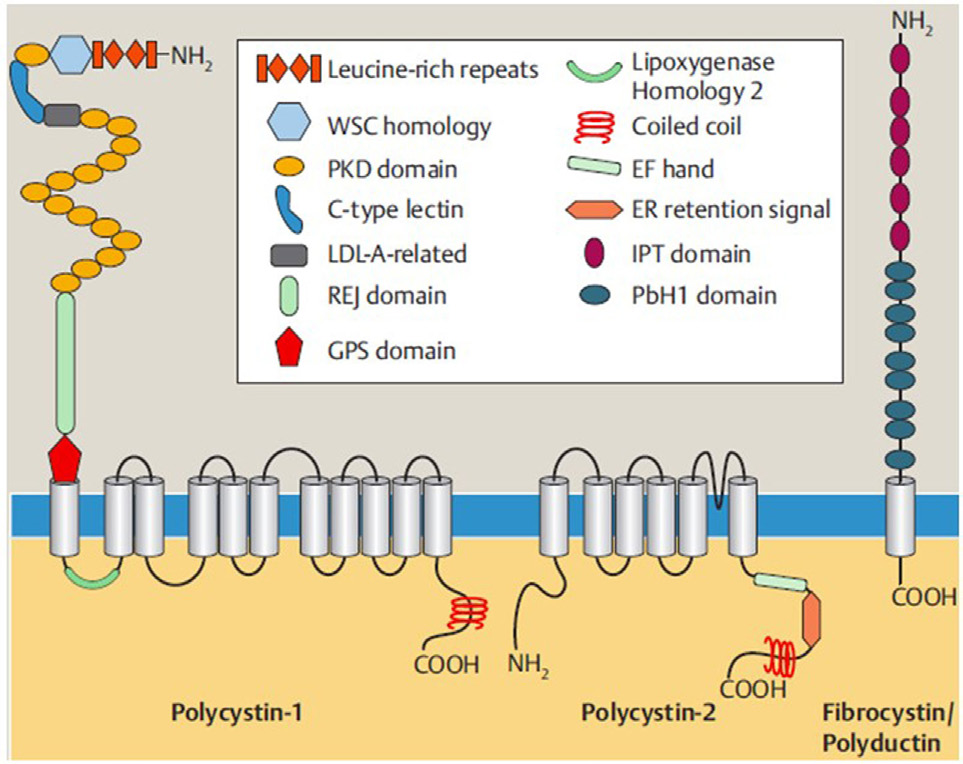
Shown are the structures of polycystin-1, polycystin-2, and fibrocystin/polyductin, the main proteins for ADPKD and ARPKD that form a protein network in polycystic kidney disease and interact with each other. Polycystin-1 and polycystin-2 are ciliary multipass transmembrane proteins, while fibrocystin/polyductin represents a type 1-membrane protein, each with an intracellular C-terminal end. The proteins’ functions are still not fully understood, but roles, e.g., as mechanoreceptor involved in cell cycle regulation, planar cell polarity, and cell–cell and cell–matrix interactions have been discussed. Polycystin-2 (also known as TRPP2) is a member of the TRP (transient receptor potential) channel family and a non-selective cation channel important for Ca^2+^-homeostasis, etc. Both for polycystin-1 and for fibrocystin/polyductin, processed isoforms in the cytoplasma and the nucleus have been described that underlie regulated intramembrane proteolysis and may serve as transcription factors [from Bergmann (33); 9:151–180].

The predicted full-length protein (termed fibrocystin or polyductin) represents a novel integral membrane protein with a signal peptide at the amino terminus of its extensive, highly glycosylated extracellular domain, a single transmembrane (TM)-spanning segment, and a short cytoplasmic C-terminal tail containing potential protein kinase A phosphorylation sites (Figure 2). Fibrocystin localizes to the ciliary membrane where it is thought to facilitate ciliary targeting signals through an 18 amino acid cytoplasmic tail (32). In line with its proposed role as a ciliary-localized membrane protein, an 18-residue motif in the cytoplasmic tail serves as a ciliary targeting signal (32). The ~3,860 amino acid extracellular portion contains several IPT and IPT-like domains similar to those found in some cell surface receptors and transcription factors. Between the IPT domains and the TM segment, multiple PbH1 repeats are present, a motif also found in polysaccharidases that may bind to carbohydrate moieties such as glycoproteins on the cell surface and/or in the basement membrane. The structural characteristics of PKHD1 and the histological defects found in human ARPKD patients suggest that fibrocystin may be involved in regulating cell–cell adhesion and proliferation. Additionally, its predicted functional domains, membrane localization, and TM characteristics suggest that it may act as a membrane-bound receptor (30).

Like polycystin-1 and polycystin-2, fibrocystin is localized to the primary cilium and basal body. During renal development, fibrocystin localizes to the apical aspect of nephronic precursors and becomes concentrated at the basal body during the early stages of ciliogenesis (12, 34–37). This spatio-temporal subcellular localization in conjunction with its interaction with polycystin-2 and CAML suggest that fibrocystin might be involved in microtubule organization and/or in mechano- or chemosensation, key functions of the primary cilia (38).

In addition, there is preliminary evidence of fibrocystin isoproteins which may be secreted in exosomes and undergo posttranslational processing (12, 34–37, 39, 40). However, the number of alternative PKHD1 transcripts that are actually translated into protein and exert biological function(s) is as yet unknown. In order to fully understand the role that individual isoforms play in the maintenance of renal and hepatobiliary function, we will need to improve our understanding of fibrocystin in the pathophysiology of ARPKD. The relatively even distribution of described mutations along the entire length of *PKHD1* suggests that the full transcript is required for proper fibrocystin function. It could be hypothesized that a certain amount of full-length functional protein is required for normal function. Alternatively, it could be the case that mutations disrupt the relative abundance of different functional isoforms of fibrocystin, which are otherwise maintained in strict stoichiometric ratios by splicing patterns.

## *PKHD1* Mutation Spectrum

The considerable size of *PKHD1*, its presumably complex splicing pattern, and our limited understanding of the protein’s function(s) present challenges to DNA-based diagnostic testing.

Further requirements for investigation are set by the extensive allelic heterogeneity with a high number of missense mutations and private mutations in non-isolate populations (19, 41–46). Thus, we decided to set up a locus-specific database for *PKHD1* that helps in the assessment of changes. Homozygous or compound heterozygous mutations in *PKHD1* are found. In approximately 80% of ARPKD patients, ranging from individuals with perinatal demise to moderately affected adults (19, 45–48). At least one mutation is even found in more than 95% of families screened. One reason for missing mutations may have been the limited sensitivity of the screening methods (e.g., SSCP, DHPLC) used in earlier studies. Moreover, silent exonic changes and intronic sequence variations may also have an effect on *PKHD1* splicing, e.g., by affecting splice enhancer or silencer sites (49). Functional and mRNA studies are usually needed to prove a possible pathogenic effect of such changes (50). Mutations may also be “hidden” in non-coding regulatory elements making them difficult to detect. In addition, the *PKHD1* gene might also be susceptible to genomic rearrangements (51). However, in patients with only one *PKHD1* variant or without any detectable *PKHD1* mutation, differential diagnoses of ARPKD should be considered. With the advance of next-generation sequencing (NGS) based testing options, many patients primarily diagnosed as ARPKD have been re-classified. It is also becoming increasingly apparent that the characteristics of ARPKD can be mimicked by mutations in a number of other genes (52). Moreover, ARPKD is not as homogeneous as widely proposed and evidence for genetic heterogeneity has been found in a subset of families.

The most common *PKHD1* mutation is c.107C>T (p.Thr36Met) in exon 3 that accounts for approximately 15–20% of mutated alleles (43). It is under debate whether this incidence is a result of a common ancestral mutation or a result of separate selective pressures. Given its frequency in certain populations, such as Central European populations, the former hypothesis cannot be dismissed. However, analysis of a multitude of unrelated families from varying ethnic groups from various haplotypes identified c.107C>T as a mutational “hotspot,” suggesting that factors outside of common ancestry likely account for at least some of the incidence of this mutation (19).

Overall, marked allelic heterogeneity characterizes the mutational spectrum in *PKHD1* with the majority of variants unique to single families in “non-isolate” populations. Given the size of the *PKHD1* gene and relative absence of mutational hotspots, conventional *PKHD1* molecular testing by Sanger sequencing is time-consuming and cumbersome (53, 54). NGS streamlines this process and makes molecular genetic diagnostics much more time- and cost-efficient (52). Moreover, as a number of genes need to be considered for PKD, NGS represents a powerful approach and allows simultaneous analysis of many genes in a single test at relatively low cost.

## Genotype–Phenotype Correlations

Establishing genotype–phenotype correlations for *PKHD1* has proved challenging. The high incidence of compound heterozygotes as well as multiple alleles makes correlations difficult to define consistently. In light of these challenges, genotype–phenotype correlations focus on types of mutations instead of the specific site of the mutation (42). Typically patients with at least two truncating mutations are severely affected and display perinatally or neonatal mortality. Patients with at least one missense mutation tend to be less severely affected and are more likely to survive the neonatal period. However, this is not always the case as some missense mutations can be as severe as truncating mutations. No significant clinical differences could be observed between patients with two missense mutations and those patients harboring a truncating mutation in trans; thus, the milder mutation obviously defines the phenotype (19).

Loss of function probably explains the uniformly early demise of patients carrying two severe truncating alleles. A critical amount of the full-length fibrocystin protein seems to be required for normal function that obviously cannot be compensated by alternative isoforms, which might be generated by re-initiation of translation at a downstream ATG codon. In contrast, missense mutations and small in-frame deletions may have more variable effects on protein function. Phenotypic diversity also reflects the variable extent to which different *PKHD1* missense mutations might compromise the function and/or abundance of the mutant protein. While some missense mutations might result in a relatively negligible effect on overall protein function leading to a milder disease course, others might result in loss of function in certain variants. Recent evidence suggests that complex transcriptional profiles may further play a role in defining the patient’s phenotype (54, 55).

While related individuals typically share a similar clinical course, about 20% of pedigrees display significant variability regarding peri/neonatal mortality between siblings with one sibling surviving to early childhood or longer (56). In families with at least one neonatal survivor, a higher proportion of 20 out of 48 sibships (42%) was observed (19). Controlling for family size, the risk for perinatal demise of any additional affected children was 37%. This is concerning from the standpoint of genetic counseling as the rate in this cohort is representative of patients in pediatric nephrology units as a whole. It is worth noting that the categorization of ARPKD as “mild,” “moderate,” and “severe” is an oversimplification. Neonatal survival is one of the principle determinates of the severity of ARPKD. Patients surviving past the neonatal period typically have much better prognosis. However, neonatal survival is influenced by a number of factors, including the extent of pulmonary hypoplasia and resultant lung disease, the availability and quality of intensive care facilities as well as awareness of risk of ARPKD by parents. Given the large degree of variability in the clinical course between siblings, it is clear that predicting the degree to which further children might be affected cannot be based on *PKHD1* genotype alone. Identification of potential modifying factors will be one of the principle goals of future research. Understanding the degree to which such modifiers play a role in ARPKD will be particularly important regarding hypomorphic missense mutations and may explain the variable clinical course associated with such cases. Conversely given the comparatively consistent clinical course in the case of null alleles modifying factors are likely to be less relevant.

## Differential Diagnosis

### DZIP1L-Related Polycystic Kidney Disease

Recently, mutations in *DZIP1L* have been described in patients with a moderate clinical course of ARPKD. DZIP1L encodes a ciliary TZ protein (DZIP1L, DAZ interacting protein 1-like) that in line with the other PKD proteins localizes to centrioles and the distal end of basal bodies. DZIP1L was demonstrated to interact with septin2 (SEPT2), a protein implicated in maintenance of the periciliary diffusion barrier at the ciliary TZ. Consistent with a defect in the diffusion barrier, the ciliary membrane translocation of the ADPKD proteins, polycystin-1 and -2, has been shown to be compromised in DZIP1L mutant cells suggesting a requirement for DZIP1L in regulating TZ integrity.

The *DZIP1L* gene is quite easy to analyze and spans only about 53 kb of genomic DNA with 16 exons (ATG start codon in exon 2) that encode a protein of 767 amino acids. Mutations in *DZIP1L* are a considerably rarer cause of ARPKD than *PKHD1* mutations, maybe due in part to the larger size of *PKHD1* relative to *DZIP1L*. Furthermore, our limited data suggest that the region encoding the N-terminus of DZIP1L may be more susceptible to mutations, a concept supported by *in silico* data that predict pathogenicity scores to decay toward the C-terminus.

The clinical course of patients and siblings with *DZIP1L* mutations described so far were invariably moderate. While the clinical manifestations in all of those patients were first detected prenatally or during early childhood, none showed perinatal demise. So far, the data suggest that the type or location of *DZIP1L* mutation does not determine the severity of the clinical course. Patients bearing missense mutations on both parental alleles displayed a comparable phenotype to patients with two truncating *DZIP1L* alleles. Although we have not screened large numbers of embryonically lethal patients, in view of homozygous truncating mutations in two of our patients we hypothesize that it is unlikely that *DZIP1L* mutations play a major role in this cohort of most severely affected patients.

### Early and Severe Autosomal Dominant Polycystic Kidney Disease (ADPKD)

**Figure 3:**
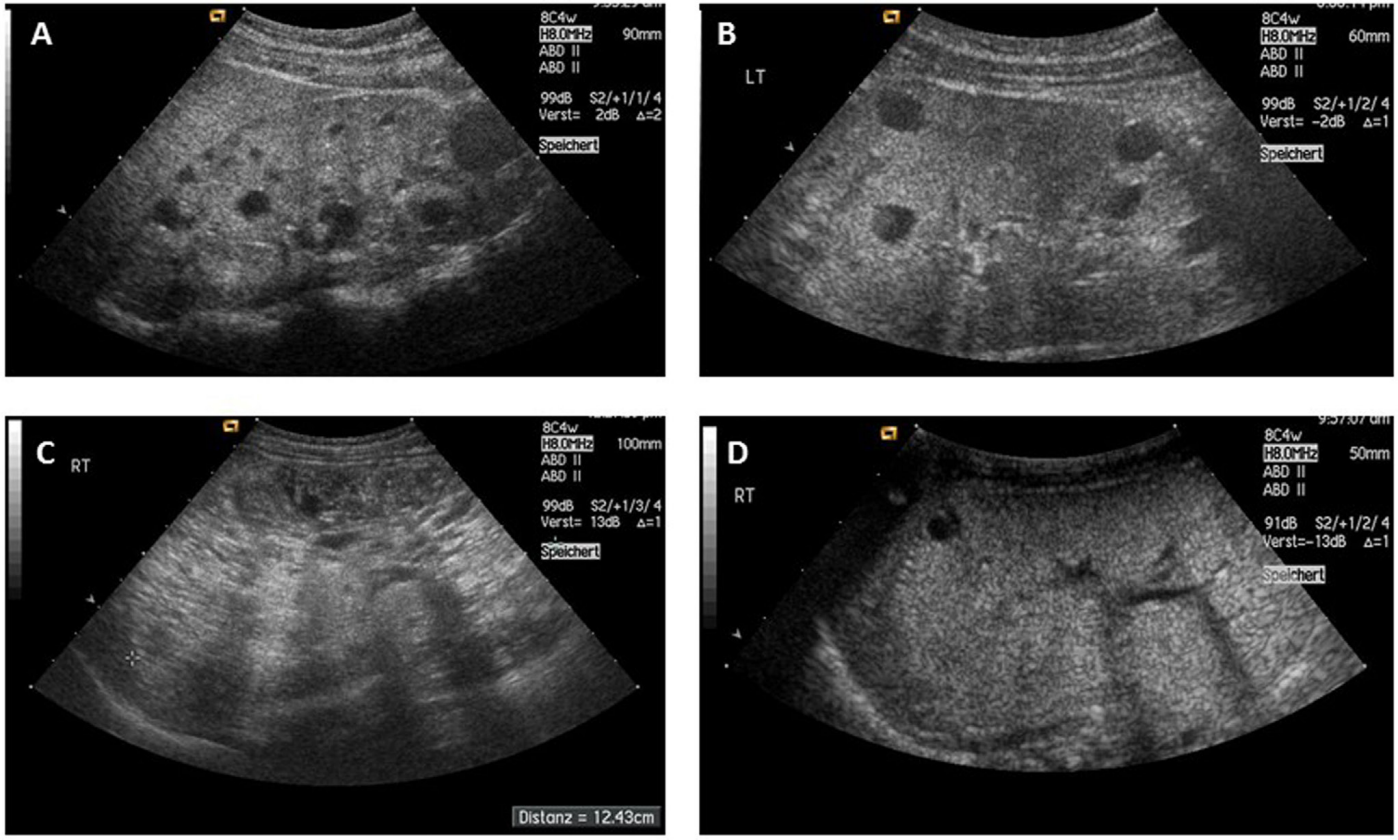
**(A,B)** Typical sonographic picture of ADPKD in a 10-month-old girl **(A)** and an 11-year-old boy **(B)**. **(C)** ADPKD resembling ARPKD in a 3-year-old boy with enlarged echogenic kidneys and small sized cysts. **(D)** Three-week-old girl with previous oligohydramnion, arterial hypertension, and small cysts in massively enlarged kidneys with a calculated total kidney volume of about 100 ml (normal age-related value <40 ml). Her mother’s phenotype resembled ADPKD, but the family was shown to carry an *HNF1β* germline mutation [from Bergmann (5)].

**Figure 4:**
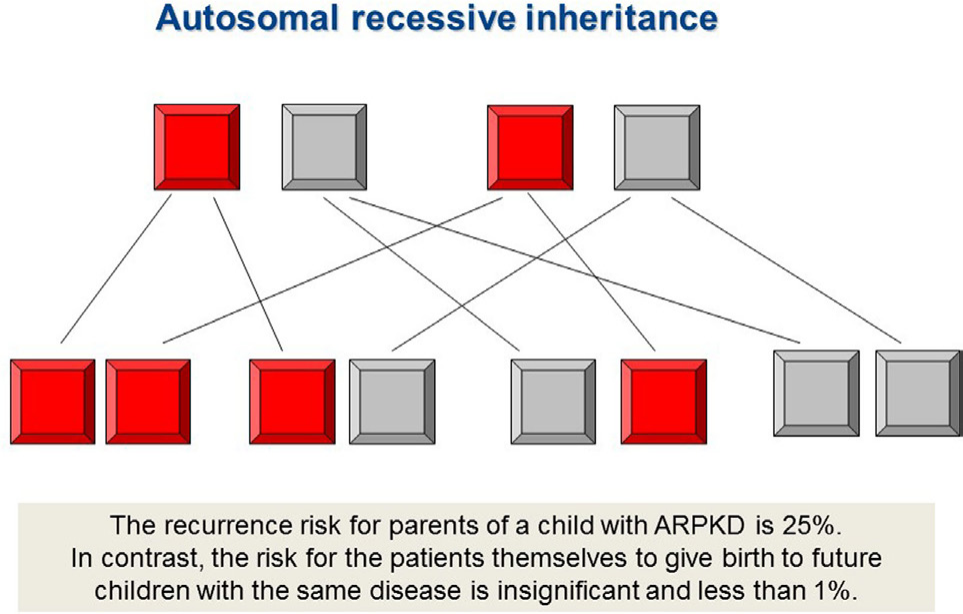
Autosomal recessive inheritance. *PKHD1* mutations and most ciliopathies are inherited in a recessive mode, however, importantly also mutations in dominant disease genes such as *PKD1* and *PKD2* can follow autosomal recessive traits. The family history usually is negative. Both, father and mother, are healthy and typically carry an “unfavorable” recessive disease allele in heterozygous state which is not sufficient to manifest the disease, however. By definition, both disease alleles need to be mutated in affected individuals with autosomal recessive disease. The recurrence risk for parents of a child with ARPKD is 25%. In contrast, the risk of patients to give birth to children with the same disease is insignificant and less than 1% in case the patient’s partner does not originate from the same family (consanguineous marriage), is not equally affected and the disease is not present in the partner’s pedigree.

**Figure 5:**
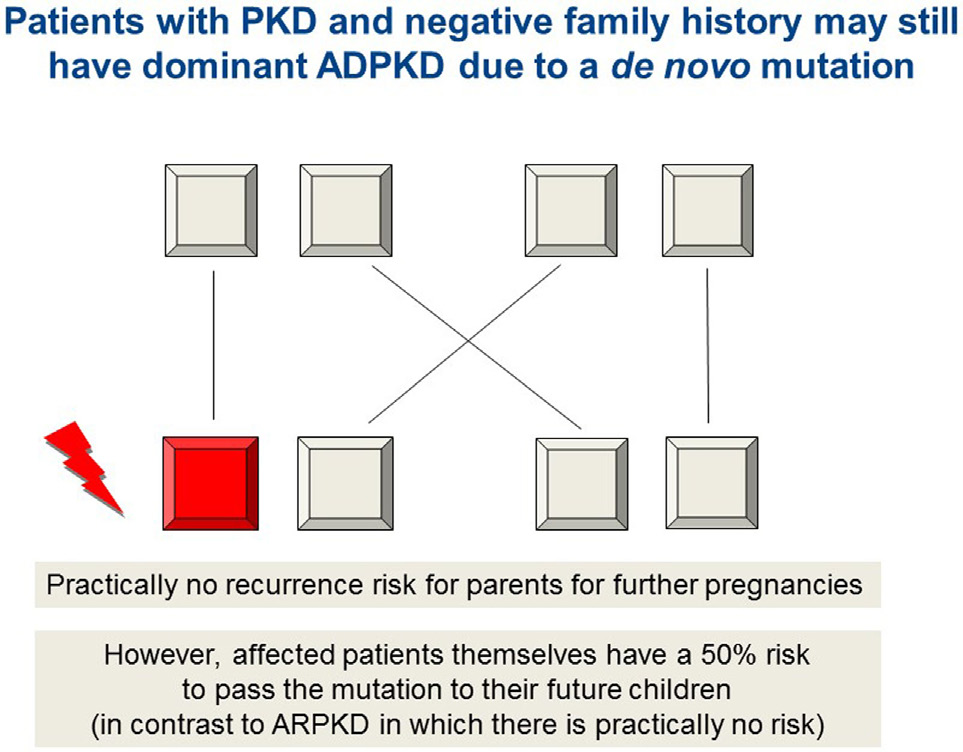
Autosomal dominant inheritance due to a *de novo* mutation in *PKD1, PKD2*, or *HNF1β*, for example, which may mimic ARPKD. As in autosomal recessive inheritance, the family history is unremarkable and both parents are healthy. For the rest of the family as well as for parents and patients, it is of major importance to know if they are afflicted by a recessive or a dominant disease due to a *de novo* arisen mutation. The latter means that there is practically no recurrence risk for future children neither for the parents nor for other family members. In contrast, the affected patients themselves bear a 50% risk to pass the (dominant) mutation to their own future children (in contrast to ARPKD in which there is practically no risk for the patients to have own children affected by the disease).

Typically, ADPKD symptoms do not become apparent before adulthood, notably, amongst a small proportion (2–5%) of ADPKD patients symptoms can manifest during childhood or even prenatally. While there is significant phenotypic variability within the same family, families in which one child displays an early manifestation of ADPKD have a high recurrence of early onset in further children suggesting a common familial background modifier that leads to early disease expression (57). Early disease manifestation in ADPKD is the most important differential diagnosis of ARPKD (Figure 3). Parental renal ultrasound is a must and should be performed in every child with cystic kidney disease of unknown origin. However, the family history often is unremarkable mainly because of two reasons: first, some alleles are incompletely penetrant (hypomorphic) and inherited in a recessive manner (58) (Figure 4). Second, a considerable proportion (about 15–20%) of all mutations in ADPKD are thought to arise *de novo* (Figure 5).

The majority of ADPKD patients carries a germline mutation in *PKD1* encoding polycystin-1, whereas about 15–20% harbor a mutation in *PKD2* leading to alterations of the polycystin-2 protein (38). PKD2 is usually significantly milder than PKD1 with development of ESRD at a later age and a lower prevalence of arterial hypertension and urinary tract infections. Patients with PKD1 develop ESRD earlier than patients with PKD2. The median age of ESRD onset in PKD1 patients is ~20 years earlier than PKD2 (58.1 and 79.9 years, respectively). Additionally, PKD1 patients present with more severe cysts than PKD2; however, this is likely due to development of cysts at an earlier age rather than an increase in cyst growth (59). Analysis of over 500 families with ADPKD revealed that patients with truncating *PKD1* mutations develop ESRD an average of 12 years earlier than those with non-truncating mutations in this gene (55.6 vs. 67.9 years) (60).

Recently, other genes such as GANAB have been described to be mutated in a subset of patients with ADPKD (61). ADPKD has a particularly high amount of allelic heterogeneity. A significant proportion of mutations are private and the majority of known *PKD1* and *PKD2* mutations are predicted to be truncating mutations (70 and 80%, respectively).

Polycystin-1 and polycystin-2 are TM proteins that interact with each other through their C-terminal tails containing coiled-coil motifs (Figure 2). Polycystin-2 is a divalent cation channel involved in cellular Ca^2+^ signaling (62). Polycystin-1 forms a complex with Polycystin-2 and, when bound to a as yet unidentified ligand, facilitates conformational changes in Polycystin-2 opening the Ca^2+^ channel. The polycystin complex is also hypothesized to be activated by mechanical strain. There is also data from functional analysis and mutations that suggest fibrocystin may also be part of the polycystin complex (63–66).

Screening for *PKD1* mutations is confounded by the six pseudogenes adjacent to the *PKD1* locus. Whether or not these pseudogenes function as junk DNA is a matter of debate. Some studies suggest that *PKD1* pseudogenes have a functional role in regulating gene expression, perhaps by acting as decoys for micro RNAs (67). In recent years, the increased sophistication of targeted NGS approaches has allowed mutational analysis of even highly confounding loci such as *PKD1* (68).

It has become clear that ADPKD is in fact a systemic disorder displaying high co-morbidity in a number of organs including the liver, pancreas, arachnoid membrane, and heart. In ADPKD patients, PLD is common and notably there appears to be evidence for a dosage sensitive network suggesting both genetic and functional overlaps between PKD and PLD (69). A broad range of cardiovascular comorbidities may also arise. Approximately 8% of ADPKD develop intracerebral aneurysms. There is also a high prevalence of cardiac valve disease in ADPKD patients with 25% of patients developing valve defects, though this is usually subclinical.

### Early and Severe PKD due to *TSC2-PKD1* Microdeletion

**Figure 6:**
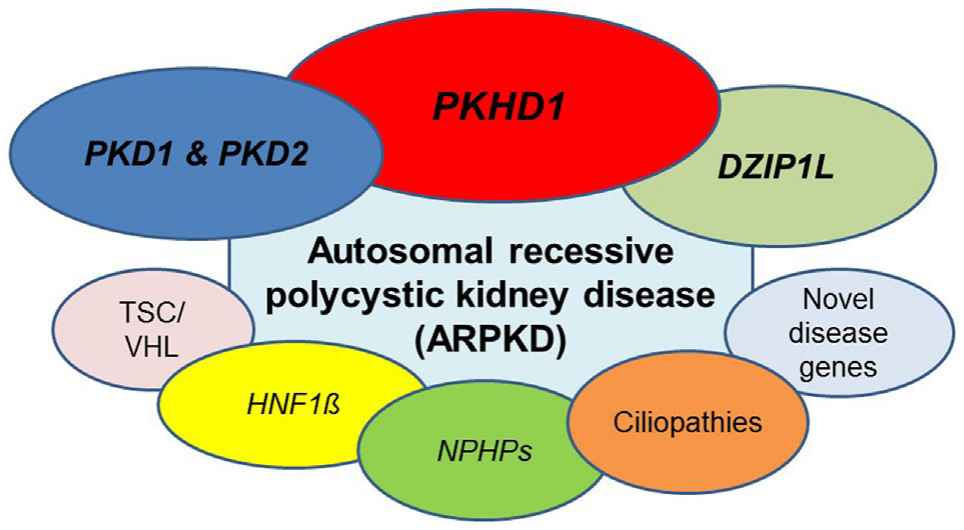
ARPKD can be caused by mutations in different genes. Beyond doubt, the main gene mutated is *PKHD1*, however, the phenotype is genetically not as homogeneous as often thought. A number of other recessive and dominant genes need to be considered. Most important are dominant and recessive mutations in *PKD1* and *PKD2*, the two genes mainly mutated in patients with adult-onset ADPKD. Other entities that may have to be discussed are *HNF1β*, novel genes for ARPKD such as *DZIP1L* and genes that typically cause other cystic kidney diseases and ciliopathies (see main text for details).

ADPKD can be mimicked by tuberous sclerosis (TSC) and von Hippel–Lindau (VHL) syndrome (Figure 6). TSC is caused by an autosomal dominant germline mutation in either *TSC1* or *TSC2* and can affect a broad range of organs with a birth incidence of approximately 1:6,000 (70). Renal manifestations are the leading cause of death in adult patients (71); cystic kidney disease occurs in 50%, angiomyolipomas are diagnosed in even 80% of patients. Tumors (mainly non-cancerous) may develop, principally in the heart and brain. Patients harboring a deletion encompassing both the *PKD1* and *TSC2* gene develop an early and severe onset of PKD.

Von Hippel–Lindau is an autosomal dominant disorder caused by mutations of the *VHL* gene. Causative mutations deactivate *VHL* which is a tumor suppressor gene, this leads to the development of hemangioblastoma in a number of CNS tissues. The CNS tumors are often accompanied by renal tumors in the form of renal clear cell carcinoma (72). VHL patients also have an increased risk of renal and pancreatic cysts.

The phenotypic overlap between TSC, VHL, and PKD patients suggests there is some functional relationship between the causative genes/proteins. From a functional standpoint, the common thread between these three entities appears to be the primary cilium and more specifically the mTOR signaling network (73). Polycystin-1 and the TSC1/TSC2 tumor suppressor complex both act to suppress the activity of mTOR. This leads to G1-cell cycle arrest resulting in apoptosis. Interestingly, it has also been demonstrated that the TSC2 protein tuberin traffics polycystin-1 to the plasma membrane (74), which may be the underlying molecular basis for the apparent genetic interaction.

### HNF1β-Related Disease

ARPKD and ADPKD are subject to a large number of phenocopies (Figures 3 and 6). One such example is HNF1β (*HNF1β/TCF2* gene). Mutations in *HNF1β* can result in a broad range of phenotypes, many of which are common in other cystic kidney diseases and ciliopathies and as such plays a critical role in the diagnostics of cystic kidney disease. *HNF1β* mutations are commonly designated as renal cyst and diabetes (75) syndrome. However, other characteristics include abnormalities of the genital tract, endocrine/exocrine insufficiency, hypomagnesemia, and an increase in liver enzymes.

*HNF1β* is inherited autosomal dominantly. As is often the case for transcription factors, there is a high degree of variability in penetrance and expressivity as well as a high frequency of spontaneous mutations which need to be considered when evaluating family history. Around 30–50% of HNF1β patients possess *de novo* mutations. Additionally, approximately 50% of patients have large deletions that remove around 1.4 Mb of genomic DNA, which includes *HNF1β* and several other genes on the 17q12 chromosome. Surprisingly, many patients carrying the microdeletion do not show a more severe phenotype than patients harboring an *HNF1β* point mutation. However, there is now convincing evidence that patients with a large genomic rearrangement in 17q12 are much more frequently affected by cognitive impairment, seizures, and other neurodevelopmental disorders (e.g., schizophrenia and autism spectrum disorders) (76, 77).

Overall, *HNF1β* mutations are thought to constitute the main cause of prenatally diagnosed bilateral hyperechogenic kidneys (78). While many of these fetuses display normal-sized kidneys often with bilateral cortical cysts and normal amniotic fluid volume, HNF1β patients may also show Potter’s sequence with oligo-/anhydramnios and massively enlarged polycystic kidneys (>+3 SD) that mimic ARPKD. In accordance, mice with an *Hnf1β* mutation showed a PKD-like phenotype with diminished *Pkhd1* expression (79). Given the role of HNF1β as a master regulator of a number of cystic kidney disease genes including *PKHD1* and *PKD2*, there is a credible genetic cause which might explain the resemblance between HNF1β and ARPKD/ADPKD patients.

### Nephronophthisis

Nephronophthisis is the general term for a heterogeneous collection of autosomal recessive cystic kidney diseases characterized by tubulointerstitial cysts and small or normal size kidneys. The size of the kidneys is usually sufficient to differentiate NPHP from ARPKD; however, in some cases, NPHP diseases can present with ARPKD-like enlarged kidneys or even Potter-like characteristics. *NPHP* disease genes have been shown to be involved in some common functional networks. Recently, we identified ANKS6 as a core component of the cystoprotein module, which links NEK8 (NPHP9) to INVS (NPHP2) and NPHP3 (80).

Characterization of the NPHP proteins has yielded considerable gains in the fundamental mechanisms involved in cystogenesis and other cilia-associated disorders. *NPHP* mutations are a significant cause of ESRD in patients under 25. The most common form of NPHP is juvenile NPHP which is characterized initially by defects in urinary concentration as well as anemia, polyuria, and polydipsia (81, 82). The most common mutations are homozygous deletions of *NPHP1*, which represent about 20–40% of all cases with juvenile NPHP. NPHP patients have normal–small size kidneys and develop cysts at the corticomedullary boundary. However, patients do not normally display cysts until they have already developed advanced chronic kidney disease. Similarly arterial hypertension does not become clinically relevant until late in the disease course. From a histological standpoint there is significant tubulointerstitial fibrosis as well as disintegration and thickening of the basement membrane (83). Similar to many cystic kidney diseases and ciliopathies, *NPHP* genes are largely pleiotropic and can result in a number of extrarenal manifestations (84).

Medullary cystic kidney disease is caused by mutations in *MUC1* and *UMOD*. Rudimentarily, it can be described as the autosomal dominant form of NPHP. Typically, it has a later onset of ESRD than the recessive forms.

### Mutations in Other Ciliary Genes May Mimic ARPKD

In rare instances, especially in the prenatal setting and during early childhood, polycystic kidney disease may be mimicked by mutations in genes that typically cause other, usually more complex ciliopathies such as Bardet–Biedl, Joubert, and Meckel syndrome (MKS).

Bardet–Biedl syndrome (BBS) is usually characterized by obesity, hypogonadism, retinal degeneration, polydactyly, mental retardation, and renal malformations. Various additional features such as hearing loss, diabetes mellitus, and other metabolic defects have also been described. Renal disease is a major cause of morbidity and mortality in BBS and can be heterogeneous in phenotype, but often presents with features of ARPKD or ADPKD with enlarged, hyperechogenic kidneys, and loss of corticomedullary differentiation. Currently, more than 20 *BBS* genes have been identified and *ALMS1*, the causative gene for Alstrom syndrome which has significant phenotypic overlap with BBS.

At the more severe end of the ciliopathy spectrum are MKS and Joubert syndrome (JS/JBTS). Like ARPKD, MKS and JBTS both affect multiple systems. However, unlike the classical cystic kidney diseases, MKS and JBTS are characterized by severe, early-onset developmental disorders rather than degenerative disorders. Consistent with many ciliopathies, they are highly heterogeneous and typically present with a host of extrarenal manifestations including hepatobiliary DPM, occipital meningoencephalocele (MKS), and postaxial polydactyly. Prognosis of patients varies considerably. Survival past the neonatal period is quite rare in MKS with a significant portion of patients dying *in utero*. MKS and JBTS also display very pronounced defects in brain development leading to mental retardation among other neural defects. The “molar tooth sign” is typical for JBTS and an indicator of underlying malformation of the mid- and hindbrain (85). MKS and JBTS patients may also display a range of characteristics common to many ciliopathies such as liver fibrosis, polydactyly and cystic kidneys as well as feeding and respiratory issues.

## Genetic Testing

In the past, strategies for genetic testing were mainly based on time- and cost-intensive single-gene testing. The advances in NGS now allow simultaneous analysis of a large group of genes in a single test at relatively low cost. It can be expected that performing single-gene testing will soon be the exception rather than the rule, especially for genetically heterogeneous disorders with a broad phenotypic spectrum such as cystic kidney disease.

At the moment, targeted NGS panel testing might be the most efficient diagnostic approach. Whatever primary strategy is chosen, it is important to be aware of the strengths and pitfalls of each method (68). Close interdisciplinary collaborations between pediatricians and clinical geneticists may prove beneficial. For polycystic kidney disease, the testing approach should be able to detect copy-number variations (CNVs) such as heterozygous deletions (e.g., in *HNF1B*) and to cover even complex genomic regions such as the *PKD1* gene. It needs to be clear that the workflow in NGS, especially with regard to complex genomic regions is much more sophisticated than for conventional approaches and requires specific expertise and infrastructure. While genetic testing evolves rapidly, there are still some limitations to consider. A significant problem many physicians in different parts of the world face is the cost of genetic testing. Although costs are decreasing due to the advances of NGS, they are still comparably high and the reason why genetics is still not used in many healthcare systems on a broader range for children with kidney diseases. Another major challenge that need to be tackled in future years in terms of personalized health efforts is a better and more precise prediction of the individual clinical course from genotype and other *in vitro* data.

Nevertheless, despite all obstacles, it is widely recommended to offer genetic testing to every family facing early-onset bilateral cystic kidney disease and to discuss the medical and ethical implications in an interdisciplinary manner. For example, genetic testing may:

– lead to earlier diagnosis and avoid a “diagnostic odyssey” and unnecessary diagnostic measures (e.g., renal or liver biopsy in patients with hyperechogenic kidneys).
– establish a definite diagnosis (“relevance to finally give the disease a name”), which represents an underestimated issue many patients and families find psychologically helpful.
– point to renal and extrarenal comorbidities which may otherwise have taken longer to diagnose and which may benefit from early detection and disease monitoring.
– highlight possible future complications which allows focused screening and better prevention.
– enable informed genetic counseling and concise information on recurrence risk for future children or other family members.

In addition, in the future, genetic diagnosis may provide guidance for personalized medical management on a more gene-specific basis (“precision medicine”). The specific genotype of a patient is particularly important when deciding to include patients in clinical trials and when choosing future treatment options. For example, individuals with hypomorphic *PKD1/PKD2* alleles are likely to have a slower disease progression and may in fact never develop ESRD. In such cases, the benefit of including the patient in clinical trials for a new treatment may not outweigh the risk.

Though there is significant overlap between ARPKD and ADPKD, there are a number of complications associated with each disease, respectively, which are relevant for proper clinical management. ADPKD patients rarely develop hepatobiliary complications like CHF or DPM. Patients with ARPKD on the other hand will almost certainly develop some complications due to CHF and DPM over time. In a similar vein, ADPKD patients require special considerations for cardiovascular comorbidities, in particular, intracerebral aneurysms (ICAs) (86) which do not typically occur in ARPKD. While general screening for ICAs is usually not recommended, it might be good to know both from the patient’s as well as from the doctor’s side whether there is a positive family history which can lead to an increased risk (87–89). Typically aneurysm rupture is quite rare in children and young adults, so screening is usually not necessary until patients reach the age of 20 years.

Many cystic kidney diseases and ciliopathies display a significant overlap of phenotypes. However, many individual diseases have particular complications that require specific clinical management. Because of the phenotypical similarity of many diseases, genomic screening is now standard practice to delineate underlying causative mutations. One example that demonstrates importance of delineating genetic causation are *NPHP* mutations. As mentioned previously in the review, NPHP is characterized by a broad range of symptoms and can, in some cases, mimic ARPKD. In addition to the characteristics it shares with ARPKD, *NPHP* mutations may often lead to retinitis pigmentosa, which is not a feature of ARPKD. Unfortunately, there is no cure for RP currently and ultimately visual impairment is inevitable. In such cases, knowing the genetic cause of the disease allows the patient and their family to prepare for such difficulties later in life. For example, pre-emptively learning braille can ease the progressive transition into visual impairment and is strongly linked to both academic and employment success. Conversely, if definitive mutations are found in other genes then the patient and their family can be assured that visual impairment is not part of the disease spectrum.

Another such example is Alstrom syndrome, which shares many common features with BBS such as retinal dystrophy and obesity. However, Alstrom syndrome patients typically have normal intelligence and do not develop polydactyly, whereas they tend to have much more severe sensorineural hearing loss as well as development of early-onset type 2 diabetes mellitus. Additionally, Alstrom syndrome patients often develop a number of cardiac, pulmonary and hepatic complications that require ongoing clinical management.

## Genetic Counseling

It is only possible to discuss in detail clinical course, disease spectrum, and recurrence risk if the nature of the patient’s genotype is known. Understandably, it is extremely important for parents who already have an affected child to know if they carry a 50% (as for autosomal dominantly inherited disorders), 25% (as for recessive diseases), or practically no recurrence risk (Figures 4 and 5). The latter holds true when the mutation in the patient arose *de novo*. A 100% guarantee cannot be given because of theoretically possible germline mosaicism in one of the parents; thus, the term “practically no recurrence risk” is usually used. In HNF1β, 30–50% of mutations arise *de novo*, while this number is thought to be 15–20% in ADPKD. In cases with a *de novo* mutation, patients carry a 50% recurrence risk for their own offspring, whereas as said it is practically zero for the parents and the rest of the family which means a huge relief for them (Figure 5). Genetic diagnosis is also pertinent to family members in the case of X-linked disorders. Genetic counseling is common for female siblings of an affected patient who may wish to know if they are a carrier or not and what the potential risk for their own children is.

With recent improvements to clinical management of PKD, many affected children who survive the neonatal period are likely to reach adulthood and may opt to have children themselves. In such cases, it is imperative for proper genetic counseling to know whether the patients’ causative mutations are dominant or recessive. Patients with dominant mutations will have a 50% recurrence risk, whereas those with recessive mutations will practically have no risk for affected children (risk <1%) except in rather unlikely cases where their partner has the same mutation in their pedigree, either through a carrier or an affected family member. For male patients, it may be worthwhile to determine if mutations are autosomal or X-linked, as there is no risk of father-son recurrence.

## Prenatal Diagnosis

Due to the early and severe manifestations of ARPKD and the high recurrence risk (25%), it is common for parents of children with ARPKD to undergo early screening by ultrasound prenatally or early postnatally. This allows for early diagnosis as well as guiding future family planning. However, examination by ultrasound may not be sufficient to detect a number of features of ARPKD such as kidney enlargement and increased echogenicity. As such, definitive prenatal diagnosis is often only reliably attainable through genetic screening. Typically prenatal diagnosis is only offered to families with a known risk of ARPKD as the procedure requires chorionic villus sampling or amniocentesis which are invasive. Another option, especially for couples with particularly high recurrence risk, is preimplantation genetic diagnosis (PGD). This process requires a significant amount of time investment as well as coordination and planning. In the future, non-invasive prenatal testing will likely become more prominent as the technology develops.

Needless to say, in the case of prenatal testing, medical and ethical implications require careful review by all attending physicians and the parents. These tests should be embedded in an ethical framework based on the principles of beneficence and respect for autonomy. Beneficence-based clinical judgment should be evidence-based, i.e., on the basis of the best available evidence, clinical strategies should be identified that are reliably expected to result in the greatest balance of benefits (i.e., the protection and promotion of health-related interests) over clinical harms (i.e., impairments of those interests) (8). Respect for autonomy and the patient’s perspective is another key issue in the context of perinatal genetics. The physician is obliged to respect parents’ values and beliefs, to respect their perspective on their interests, and to implement only those clinical strategies authorized as the result of the informed consent process (8).

## What have We Learned During Recent Years and What is on the Horizon?

Considerable progress has been made in the understanding of polycystic kidney diseases, including ARPKD. However, there are still more questions than answers and we wonder about phenomena such as significantly variable disease expression even within the same family. Stochastic, epigenetic, and environmental factors can be expected to modify the phenotype. While standard Mendelian genetics will explain the expressivity of disease in many patients, a significant portion will have more complex genotypes often leading to more severe expression of disease (2). Factors such as second-site modifying alleles may have an epistatic effect and contribute to increased severity. Additionally, hypomorphic accessory mutations may lead to the reduction in the overall dose of function proteins and disrupt so called “dosage sensitive network” leading to a more severe clinical outcome.

Given the considerable heterogeneity, prevalence of phenocopies and overall similarity between many cystic kidney diseases and ciliopathies the possible causative gene (or genes) may number in hundreds (Figure 6). In order to analyze all the potential disease genes in a given case, clinicians now routinely utilize NGS techniques. In addition, NGS also allows for the identification of features such as CNVs, unlike Sanger sequencing. Identification of deletions/duplications is vital as they represent about 5% of the mutational spectrum for some genes. In some case such as HNF1β deletions represent large proportion (around 50%) of all causative mutations.

Overall, the high-throughput targeted analysis of patients’ genomes allows for accurate genetic diagnosis of disease and helps to avoid misdiagnosis. Additionally, increased usage of NGS allows clinicians and researchers to better interpretation identified variants. For the time being, targeted NGS panel approaches are considered as the method of choice for cystic and polycystic kidney diseases considerably increasing the detection rates. However, genetic technologies are evolving rapidly and whole-genome sequencing (WGS) offers advantages over other methodologies and will likely be used in greater volumes for diagnostic purposes. In future years, WGS may eventually even serve as a universal first line test for any disorder with suspected genetic origin. However, currently a one-size-fits-all approach is definitely not the best approach. Next-generation sequencing (NGS) has revolutionized genetic diagnostics and already became a commodity that helps to provide rapidly growing insight into the discussed issues. Interpretation of data becomes the challenge and bench and bedside benefit from digitized medicine and smart bioinformatics algorithms embedded in a multidisciplinary setting.

## Author Contributions

The author confirms being the sole contributor of this work and approved it for publication.

## Conflict of Interest Statement

The author declares that the research was conducted in the absence of any commercial or financial relationships that could be construed as a potential conflict of interest.

## References

[1] Fliegauf M Benzing T Omran H. When cilia go bad: cilia defects and ciliopathies. Nat Rev Mol Cell Biol (2007) 8:880–93. 10.1038/nrm2278 17955020

[2] Gerdes JM Davis EE Katsanis N. The vertebrate primary cilium in development, homeostasis, and disease. Cell (2009) 137:32–45. 10.1016/j.cell.2009.03.023 19345185 PMC3016012

[3] Nigg EA Raff JW Centrioles, centrosomes, and cilia in health and disease. Cell (2009) 139:134 10.1016/j.cell.2009.10.036 19914163

[4] Hildebrandt F Benzing T Katsanis N Ciliopathies. N Engl J Med (2011) 364:1533–43. 10.1056/NEJMra1010172 21506742 PMC3640822

[5] Bergmann C ARPKD and early manifestations of ADPKD: the original polycystic kidney disease and phenocopies. Pediatr Nephrol (2015) 30(1):15–30. 10.1007/s00467-013-2706-2 PMC4240914 24584572

[6] Sweeney WE Jr Avner ED. Diagnosis and management of childhood polycystic kidney disease. Pediatr Nephrol (2011) 26:675–92. 10.1007/s00467-010-1656-1 21046169

[7] Drenth JP Chrispijn M Bergmann C. Congenital fibrocystic liver diseases. Best Pract Res Clin Gastroenterol (2010) 24:573–84. 10.1016/j.bpg.2010.08.007 20955960

[8] Bergmann C Weiskirchen R It’s not all in the cilium, but on the road to it: genetic interaction network in polycystic kidney and liver diseases and how trafficking and quality control matter. J Hepatol (2012) 56:1201–3. 10.1016/j.jhep.2011.10.014 22133568

[9] Jensen VL Li C Bowie RV Clarke L Mohan S Blacque OE Formation of the transition zone by Mks5/Rpgrip1L establishes a ciliary zone of exclusion (CIZE) that compartmentalises ciliary signalling proteins and controls PIP2 ciliary abundance. EMBO J (2015) 34:2537–56. 10.15252/embj.201488044 26392567 PMC4609185

[10] Reiter JF Blacque OE Leroux MR. The base of the cilium: roles for transition fibres and the transition zone in ciliary formation, maintenance and compartmentalization. EMBO Rep (2012) 13:608–18. 10.1038/embor.2012.73 22653444 PMC3388784

[11] Guay-Woodford LM. Autosomal recessive polycystic kidney disease: the prototype of the hepato-renal fibrocystic diseases. J Pediatr Genet (2014) 3:89–101. 10.3233/PGE-14092 25632369 PMC4306463

[12] Wang S Luo Y Wilson PD Witman GB Zhou J. The autosomal recessive polycystic kidney disease protein is localized to primary cilia, with concentration in the basal body area. J Am Soc Nephrol (2004) 15:592–602. 10.1097/01.ASN.0000113793.12558.1D 14978161

[13] Liebau MC Bergmann C In: Geary DF Schaefer F, editors. Polycystic Kidney Disease: ADPKD and ARPKD. Pediatric Kidney Disease. Berlin, Heidelberg: Springer (2016).

[14] Bergmann C Küpper F Dornia C Schneider F Senderek J Zerres K Algorithm for efficient PKHD1 mutation screening in autosomal recessive polycystic kidney disease (ARPKD). Hum Mutat (2005) 25:225–31. 10.1002/humu.20145 15706593

[15] Guay-Woodford LM Desmond RA. Autosomal recessive polycystic kidney disease: the clinical experience in North America. Pediatrics (2003) 111:1072–80. 10.1542/peds.111.5.1072 12728091

[16] Gunay-Aygun M Avner ED Bacallao RL Choyke PL Flynn JT Germino GG Autosomal recessive polycystic kidney disease and congenital hepatic fibrosis: summary statement of a first National Institutes of Health/Office of Rare Diseases Conference. J Pediatr (2006) 149:159–64. 10.1016/j.jpeds.2006.03.014 16887426 PMC2918414

[17] Kääriäinen H. Polycystic kidney disease in children: a genetic and epidemiological study of 82 Finnish patients. J Med Genet (1987) 24:474–81. 10.1136/jmg.24.8.474 3656369 PMC1050204

[18] Adeva M El-Youssef M Rossetti S Kamath PS Kubly V Consugar MB Clinical and molecular characterization defines a broadened spectrum of autosomal recessive polycystic kidney disease (ARPKD). Medicine (Baltimore) (2006) 85(1):1–21. 10.1097/01.md.0000200165.90373.9a 16523049

[19] Bergmann C Senderek J Windelen E Clinical consequences of PKHD1 mutations in 164 patients with autosomal recessive polycystic kidney disease (ARPKD). Kidney Int (2005) 67:829–48. 10.1111/j.1523-1755.2005.00148.x 15698423

[20] Avni FE Guissard G Hall M Janssen F DeMaertelaer V Rypens F. Hereditary polycystic kidney diseases in children: changing sonographic patterns through childhood. Pediatr Radiol (2002) 32:169–74. 10.1007/s00247-001-0624-0 12164348

[21] Turkbey B Ocak I Daryanani K Autosomal recessive polycystic kidney disease and congenital hepatic fibrosis (ARPKD/CHF). Pediatr Radiol (2009) 39:100–11. 10.1007/s00247-008-1064-x 19089418 PMC2918426

[22] Desmet VJ Ludwig symposium on biliary disorders-part I. Pathogenesis of ductal plate abnormalities. Mayo Clin Proc (1998) 73:80–9. 10.4065/73.1.80 9443684

[23] Telega G Cronin D Avner ED. New approaches to the autosomal recessive polycystic kidney disease patient with dual kidney-liver complications. Pediatr Transplant (2013) 17:328–35. 10.1111/petr.12076 23593929 PMC3663883

[24] Rossetti S Torra R Coto E Consugar M Kubly V Malaga S A complete mutation screen of *PKHD1* in autosomal recessive polycystic kidney pedigrees. Kidney Int (2003) 64:391–403. 10.1046/j.1523-1755.2003.00111.x 12846734

[25] Gallagher AR Esquivel EL Briere TS Tian X Mitobe M Menezes LF Biliary and pancreatic dysgenesis in mice harboring a mutation in Pkhd1. Am J Pathol (2008) 172:417–29. 10.2353/ajpath.2008.070381 18202188 PMC2312372

[26] Garcia-Gonzalez MA Menezes LF Piontek KB Kaimori J Huso DL Watnick T Genetic interaction studies link autosomal dominant and recessive polycystic kidney disease in a common pathway. Hum Mol Genet (2007) 16:1940–50. 10.1093/hmg/ddm141 17575307 PMC2085232

[27] Moser M Matthiesen S Kirfel J A mouse model for cystic biliary dysgenesis in autosomal recessive polycystic kidney disease (ARPKD). Hepatology (2005) 41:1113–21. 10.1002/hep.20655 15830394

[28] Woollard JR Punyashtiti R Richardson S Masyuk TV Whelan S Huang BQ A mouse model of autosomal recessive polycystic kidney disease with biliary duct and proximal tubule dilatation. Kidney Int (2007) 72:328–36. 10.1038/sj.ki.5002294 17519956

[29] Ward CJ Hogan MC Rossetti S Walker D Sneddon T Wang X The gene mutated in autosomal recessive polycystic kidney disease encodes a large, receptor-like protein. Nat Genet (2002) 30:259–69. 10.1038/ng833 11919560

[30] Onuchic LF Furu L Nagasawa Y Hou X Eggermann T Ren Z PKHD1, the polycystic kidney and hepatic disease 1 gene, encodes a novel large protein containing multiple immunoglobulin-like plexin-transcription-factor domains and parallel beta-helix 1 repeats. Am J Hum Genet (2002) 70:1305–17. 10.1086/340448 11898128 PMC447605

[31] Nagasawa Y Matthiesen S Onuchic LF Hou X Bergmann C Esquivel E Identification and characterization of Pkhd1, the mouse orthologue of the human ARPKD gene. J Am Soc Nephrol (2002) 13:2246–58. 10.1097/01.ASN.0000030392.19694.9D 12191969

[32] Follit JA Li L Vucica Y Pazour GJ. The cytoplasmic tail of fibrocystin contains a ciliary targeting sequence. J Cell Biol (2010) 188:21–8. 10.1083/jcb.200910096 20048263 PMC2812845

[33] Bergmann C Zystische Nierenerkrankungen und Ziliopathien. Pädiatrie up2date (2014) 9:151–80. 10.1055/s-0034-1365722

[34] Masyuk TV Huang BQ Ward CJ Masyuk AI Yuan D Splinter PL Defects in cholangiocyte fibrocystin expression and ciliary structure in the PCK rat. Gastroenterology (2003) 125:1303–10. 10.1016/j.gastro.2003.09.001 14598246

[35] Menezes LF Cai Y Nagasawa Y Silva AM Watkins ML Da Silva AM Polyductin, the PKHD1 gene product, comprises isoforms expressed in plasma membrane, primary cilium, and cytoplasm. Kidney Int (2004) 66:1345–55. 10.1111/j.1523-1755.2004.00844.x 15458427

[36] Ward CJ Yuan D Masyuk TV Wang X Punyashthiti R Whelan S Cellular and subcellular localization of the ARPKD protein; fibrocystin is expressed on primary cilia. Hum Mol Genet (2003) 12:2703–10. 10.1093/hmg/ddg274 12925574

[37] Zhang MZ Mai W Li C Cho SY Hao C Moeckel G PKHD1 protein encoded by the gene for autosomal recessive polycystic kidney disease associates with basal bodies and primary cilia in renal epithelial cells. Proc Natl Acad Sci U S A (2004) 101:2311–6. 10.1073/pnas.0400073101 14983006 PMC356947

[38] Harris PC Torres VE Polycystic kidney disease. Annu Rev Med (2009) 60:321–37. 10.1146/annurev.med.60.101707.125712 18947299 PMC2834200

[39] Hiesberger T Gourley E Erickson A Koulen P Ward CJ Masyuk TV Proteolytic cleavage and nuclear translocation of fibrocystin is regulated by intracellular Ca2+ and activation of protein kinase C. J Biol Chem (2006) 281:34357–64. 10.1074/jbc.M606740200 16956880

[40] Kaimori JY Nagasawa Y Menezes LF Garcia-Gonzalez MA Deng J Imai E Polyductin undergoes notch-like processing and regulated release from primary cilia. Hum Mol Genet (2007) 16:942–56. 10.1093/hmg/ddm039 17470460 PMC1955383

[41] Rossetti S Chauveau D Walker D Saggar-Malik A Winearls CG Torres VE A complete mutation screen of the ADPKD genes by DHPLC. Kidney Int (2002) 61:1588–99. 10.1046/j.1523-1755.2002.00326.x 11967008

[42] Bergmann C Senderek J Sedlacek B Pegiazoglou I Puglia P Eggermann T Spectrum of mutations in the gene for autosomal recessive polycystic kidney disease (ARPKD/PKHD1). J Am Soc Nephrol (2003) 14:76–89. 10.1097/01.ASN.0000039578.55705.6E 12506140

[43] Bergmann C Senderek J Küpper F Schneider F Dornia C Windelen E PKHD1 mutations in autosomal recessive polycystic kidney disease (ARPKD). Hum Mutat (2004) 23:453–63. 15108277 10.1002/humu.20029

[44] Bergmann C Senderek J Schneider F Dornia C Küpper F Eggermann T PKHD1 mutations in families requesting prenatal diagnosis for autosomal recessive polycystic kidney disease (ARPKD). Hum Mutat (2004) 23:487–95. 10.1002/humu.20019 15108281

[45] Furu L Onuchic LF Gharavi A Hou X Esquivel EL Nagasawa Y Milder presentation of recessive polycystic kidney disease requires presence of amino acid substitution mutations. J Am Soc Nephrol (2003) 14:2004–14. 10.1097/01.ASN.0000078805.87038.05 12874454

[46] Losekoot M Haarloo C Ruivenkamp C White SJ Breuning MH Peters DJ. Analysis of missense variants in the PKHD1-gene in patients with autosomal recessive polycystic kidney disease (ARPKD). Hum Genet (2005) 118:185–206. 10.1007/s00439-005-0027-7 16133180

[47] Gunay-Aygun M Tuchman M Font-Montgomery E Lukose L Edwards H Garcia A PKHD1 sequence variations in 78 children and adults with autosomal recessive polycystic kidney disease and congenital hepatic fibrosis. Mol Genet Metab (2010) 99:160–73. 10.1016/j.ymgme.2009.10.010 19914852 PMC2818513

[48] Gunay-Aygun M Font-Montgomery E Lukose L Tuchmann M Graf J Bryant JC Correlation of kidney function, volume and imaging findings, and PKHD1 mutations in 73 patients with autosomal recessive polycystic kidney disease. Clin J Am Soc Nephrol (2010) 5:972–84. 10.2215/CJN.07141009 20413436 PMC2879301

[49] Baralle D Baralle M. Splicing in action: assessing disease causing sequence changes. J Med Genet (2005) 42:737–48. 10.1136/jmg.2004.029538 16199547 PMC1735933

[50] Bergmann C Frank V Küpper F Schmidt C Senderek J Zerres K. Functional analysis of PKHD1 splicing in autosomal recessive polycystic kidney disease. J Hum Genet (2006) 51:788–93. 10.1007/s10038-006-0022-4 16897190

[51] Bergmann C Küpper F Schmitt CP Vester U Neuhaus TJ Senderek J Multi-exon deletions of the PKHD1 gene cause autosomal recessive polycystic kidney disease (ARPKD). J Med Genet (2005) 42:e63. 10.1136/jmg.2005.032318 16199545 PMC1735935

[52] Bergmann C Autosomal Recessive Polycystic Kidney Disease. Oxford: Oxford University Press (2014).

[53] Krall P Pineda C Ruiz P Ejarque L Vendrell T Camacho JA Cost-effective PKHD1 genetic testing for autosomal recessive polycystic kidney disease. Pediatr Nephrol (2013) 29(2):223–34. 10.1007/s00467-013-2657-7 24162162

[54] Boddu R Yang C O’Connor AK Hendrickson RC Boone B Cui X Intragenic motifs regulate the transcriptional complexity of Pkhd1/PKHD1. J Mol Med (2014) 92:1045–56. 10.1007/s00109-014-1185-7 24984783 PMC4197071

[55] Frank V Zerres K Bergmann C. Transcriptional complexity in autosomal recessive polycystic kidney disease. Clin J Am Soc Nephrol (2014) 9:1729–36. 10.2215/CJN.00920114 25104275 PMC4186505

[56] Deget F Rudnik-Schoneborn S Zerres K. Course of autosomal recessive polycystic kidney disease (ARPKD) in siblings: a clinical comparison of 20 sibships. Clin Genet (1995) 47:248–53. 10.1111/j.1399-0004.1995.tb04305.x 7554350

[57] Torres VE Harris PC Pirson Y. Autosomal dominant polycystic kidney disease. Lancet (2007) 369:1287–301. 10.1016/S0140-6736(07)60601-1 17434405

[58] Lu H Galeano MCR Ott E Kaeslin G Kausalya PJ Kramer C Mutations in DZIP1L, which encodes a ciliary-transition zone protein, cause autosomal recessive polycystic kidney disease. Nat Genet (2017) 49:1025–34. 10.1038/ng.3871 28530676 PMC5687889

[59] Harris PC Bae KT Rossetti S Torries VE Grantham JJ Chapman AB Cyst number but not the rate of cystic growth is associated with the mutated gene in autosomal dominant polycystic kidney disease. J Am Soc Nephrol (2006) 17:3013–9. 10.1681/ASN.2006080835 17035604

[60] Cornec-Le Gall E Audrézet MP Chen JM Hourmant M Morin MP Perrichot R Type of PKD1 mutation influences renal outcome in ADPKD. J Am Soc Nephrol (2013) 24:1006–13. 10.1681/ASN.2012070650 23431072 PMC3665389

[61] Porath B Gainullin VG Cornec-Le Gall E Dillinger EK Heyer CM Hopp K Mutations in GANAB, encoding the glucosidase iialpha subunit, cause autosomal-dominant polycystic kidney and liver disease. Am J Hum Genet (2016) 98:1193–207. 10.1016/j.ajhg.2016.05.004 27259053 PMC4908191

[62] Torres VE Harris PC. Autosomal dominant polycystic kidney disease: the last 3 years. Kidney Int (2009) 76:149–68. 10.1038/ki.2009.128 19455193 PMC2812475

[63] Bergmann C von Bothmer J Ortiz Brüchle N Venghaus A Frank V Fehrenbach H Mutations in multiple PKD genes may explain early and severe polycystic kidney disease. J Am Soc Nephrol (2011) 22:2047–56. 10.1681/ASN.2010101080 22034641 PMC3279997

[64] Kim I Fu Y Hui K Moeckel G Mai W Li C Fibrocystin/polyductin modulates renal tubular formation by regulating polycystin-2 expression and function. J Am Soc Nephrol (2008) 19:455–68. 10.1681/ASN.2007070770 18235088 PMC2391052

[65] Wang S Zhang J Nauli SM Li X Starremans PG Luo Y Fibrocystin/polyductin, found in the same protein complex with polycystin-2, regulates calcium responses in kidney epithelia. Mol Cell Biol (2007) 27:3241–52. 10.1128/MCB.00072-07 17283055 PMC1899915

[66] Wu Y Dai XQ Li Q Chen CX Mai W Hussain Z Kinesin-2 mediates physical and functional interactions between polycystin-2 and fibrocystin. Hum Mol Genet (2006) 15:3280–92. 10.1093/hmg/ddl404 17008358

[67] Poliseno L Salmena L Zhang J Carver B Haveman WJ Pandolfi PP A coding-independent function of gene and pseudogene mRNAs regulates tumour biology. Nature (2010) 465:1033–8. 10.1038/nature09144 20577206 PMC3206313

[68] Eisenberger T Decker C Hiersche M Hamann RC Decker E Neuber S An efficient and comprehensive strategy for genetic diagnostics of polycystic kidney disease. PLoS One (2015) 10:e0116680. 10.1371/journal.pone.0116680 25646624 PMC4315576

[69] Fedeles SV Tian X Gallagher AR Mitobe M Nishio S Lee SH A genetic interaction network of five genes for human polycystic kidney and liver diseases defines polycystin-1 as the central determinant of cyst formation. Nat Genet (2011) 43:639–47. 10.1038/ng.860 21685914 PMC3547075

[70] Crino PB Nathanson KL Henske EP The tuberous sclerosis complex. N Engl J Med (2006) 355:1345–56. 10.1056/NEJMra055323 17005952

[71] Shepherd CW Gomez MR Lie JT Crowson CS. Causes of death in patients with tuberous sclerosis. Mayo Clin Proc (1991) 66:792–6. 10.1016/S0025-6196(12)61196-3 1861550

[72] Kaelin WG Jr. The von Hippel-Lindau tumour suppressor protein: O2 sensing and cancer. Nat Rev Cancer (2008) 8:865–73. 10.1038/nrc2502 18923434

[73] Huber TB Walz G Kuehn EW mTOR and rapamycin in the kidney: signaling and therapeutic implications beyond immunosuppression. Kidney Int (2010) 79:502–11. 10.1038/ki.2010.457 21085109

[74] Shillingford JM Murcia NS Larson CH Low SH Hedgepeth R Brown N The mTOR pathway is regulated by polycystin-1, and its inhibition reverses renal cystogenesis in polycystic kidney disease. Proc Natl Acad Sci U S A (2006) 103:5466–71. 10.1073/pnas.0509694103 16567633 PMC1459378

[75] Verhave JC Bech AP Wetzels JF Nijenhuis T Hepatocyte nuclear factor 1beta-associated kidney disease: more than renal cysts and diabetes. J Am Soc Nephrol (2016) 27:345–53. 10.1681/ASN.2015050544 26319241 PMC4731131

[76] Moreno-De-Luca D SGENE Consortium Mulle JG Simons Simplex Collection Genetics Consortium Kaminsky EB Sanders SJ Deletion 17q12 is a recurrent copy number variant that confers high risk of autism and schizophrenia. Am J Hum Genet (2010) 87:618–30. 10.1016/j.ajhg.2010.10.004 21055719 PMC2978962

[77] Nagamani SC Erez A Shen J Li C Roeder E Cox S Clinical spectrum associated with recurrent genomic rearrangements in chromosome 17q12. Eur J Hum Genet (2010) 18:278–84. 10.1038/ejhg.2009.174 19844256 PMC2987224

[78] Decramer S Parant O Beaufils S Clauin S Guillou C Kessler S Anomalies of the TCF2 gene are the main cause of fetal bilateral hyperechogenic kidneys. J Am Soc Nephrol (2007) 18:923–33. 10.1681/ASN.2006091057 17267738

[79] Hiesberger T Bai Y Shao X McNally BT Sinclair AM Tian X Mutation of hepatocyte nuclear factor-1beta inhibits Pkhd1 gene expression and produces renal cysts in mice. J Clin Invest (2004) 113:814–25. 10.1172/JCI200420083 15067314 PMC362119

[80] Hoff S Halbritter J Epting D Frank V Nguyen TM van Reeuwijk J ANKS6 is a central component of a nephronophthisis module linking NEK8 to INVS and NPHP3. Nat Genet (2013) 45:951–6. 10.1038/ng.2681 23793029 PMC3786259

[81] Hildebrandt F Zhou W Nephronophthisis-associated ciliopathies. J Am Soc Nephrol (2007) 18:1855–71. 10.1681/ASN.2006121344 17513324

[82] Salomon R Saunier S Niaudet P. Nephronophthisis. Pediatr Nephrol (2009) 24:2333–44. 10.1007/s00467-008-0840-z 18607645 PMC2770134

[83] Omran H Nephronophthis and medullary cystic kidney disease. In: Geary DF Schaefer F, editors. Comprehensive Pediatric Nephrology. Amsterdam: Elsevier (2008). p. 143–54.

[84] Bergmann C Fliegauf M Brüchle NO Frank V Olbrich H Kirschner J Loss of nephrocystin-3 function can cause embryonic lethality, Meckel-Gruber-like syndrome, situs inversus, and renal-hepatic-pancreatic dysplasia. Am J Hum Genet (2008) 82:959–70. 10.1016/j.ajhg.2008.02.017 18371931 PMC2427297

[85] Parisi MA. Clinical and molecular features of Joubert syndrome and related disorders. Am J Med Genet C Semin Med Genet (2009) 151C:326–40. 10.1002/ajmg.c.30229 19876931 PMC2797758

[86] Xu HW Yu SQ Mei CL Li MH. Screening for intracranial aneurysm in 355 patients with autosomal-dominant polycystic kidney disease. Stroke (2011) 42:204–6. 10.1161/STROKEAHA.110.578740 21164130

[87] Pirson Y Chauveau D Torres V Management of cerebral aneurysms in autosomal dominant polycystic kidney disease. J Am Soc Nephrol (2002) 13:269–76. 11752048 10.1681/ASN.V131269

[88] Huston J III Torres VE Sulivan PP Offord KP Wiebers DO. Value of magnetic resonance angiography for the detection of intracranial aneurysms in autosomal dominant polycystic kidney disease. J Am Soc Nephrol (1993) 3:1871–7. 8338918 10.1681/ASN.V3121871

[89] Ruggieri PM Poulos N Masaryk TJ Ross JS Obuchowski NA Awad IA Occult intracranial aneurysms in polycystic kidney disease: screening with MR angiography. Radiology (1994) 191:33–9. 10.1148/radiology.191.1.8134594 8134594

